# The Role of Vitamin D, Platelet-Derived Growth Factor and Insulin-Like Growth Factor 1 in the Progression of Thyroid Diseases

**DOI:** 10.31557/APJCP.2020.21.7.2083

**Published:** 2020-07

**Authors:** Mona S Abdellateif, Sabry Shaarawy, Yasmine F Elesawy, Mona Mansour, Effat Tharwat, Noha H Ibrahim, Marwa S Eissa

**Affiliations:** 1 *Medical Biochemistry and Molecular Biology, Department of Cancer Biology, National Cancer Institute, Cairo University, Cairo, Egypt. *; 2 *Department of Pathology, Faculty of Medicine, Cairo University, Cairo, Egypt. *; 3 *Department of Internal Medicine and Endocrinology, Faculty of Medicine, Cairo University, Cairo, Egypt. *; 4 *Department of Clinical and Chemical Pathology, National Cancer Institute, Cairo University, Cairo, Egypt.*

**Keywords:** Thyroid cancer, papillary thyroid carcinoma, Vit D, PDGF, IGF-1

## Abstract

**Background:**

Thyroid cancer (TC) is a common malignant tumor, however the role of total vitamin D: 25(OH)D, Platelet Derived Growth Factor (PDGF) and Insulin Like Growth Factor 1 (IGF-1) in the development of TC is still unclear.

**Aim:**

To assess the roles of 25(OH)D, PDGF and IGF-1 in the progression of thyroid diseases. METHODS: The serum levels of 25(OH)D, PDGF and IGF-1 were assessed in 70 patients with papillary thyroid cancer (PTC), 60 patients with benign thyroid nodules (BN) compared to 60 normal controls (NC) using ELISA technique.

**Results:**

There was a significant decrease in the serum level of 25(OH)D in TC patients compared to NC (P<0.001) and BN patients (P=0.006). There was a significant increase in the serum levels of PDGF and IGF-1 in TC patients (P<0.001), and BN patients (P<0.001) compared to NC, while there were no significant differences between TC and BN (P=0.087, and 0.258; respectively). PDGF correlated significantly with IGF-1 (r=0.412, P<0.001), TSH (r=0.146, P=0.045), and inversely correlated with 25(OH)D (r= -0.156, P=0.013) and FT4 (r=-0.178, P=0.014). There was a significant inverse correlation between the serum levels of IGF-1 and FT4 (r=-0.172, P=0.017). Sensitivity and specificity for assessment of TC patients were (65.7% and 58.3%, P= 0.001) for 25(OH)D, (65.7% and 58.3%, P=0.021) for IGF-1, and (68.6% and 61.7%, P=0.006) for PDGF. Multivariate analysis demonstrated that serum 25(OH)D (OR=0.578, 95%CI= 0.426-0.783), IGF-1 (OR=1.019, 95%CI= 1.010-1.029) and PDGF (OR=1.007, 95%CI= 1.004-1.009) were considered independent risk factors for thyroid cancer (P<0.001, for all).

**Conclusion:**

25(OH) D, IGF-1 and PDGF are significantly different in TC and BN cases compared to control. They have an important role in the progression of TC. However, these data should be validated on a larger sample size.

## Introduction

Thyroid cancer (TC) is the most common malignant endocrine tumor worldwide, and it ranks the ninth in tumor incidence. Thyroid cancer occurs most commonly in females as its incidence is 3 times higher than that in males, and it accounts for 5.1% of the total estimated cancer burden in women in 2018 (Bray et al., 2018). Most thyroid cancers originate in the follicular epithelium, and it divided pathologically into three subtypes; papillary thyroid cancer (PTC), follicular thyroid cancer (FTC) and anaplastic thyroid cancer (ATC) which represent about 80%, 15% and 5% of all TC patients, respectively (Handkiewicz-Junak et al., 2010). Although most PTC and FTC cases have a favorable prognosis, however about 20–30% of PTC patients develop recurrence and reduced survival rates (Molinaro et al., 2017). Therefore, it is important to investigate the underlying mechanisms for the development and progression of TC, this will help in identifying potential biomarkers that could differentiate various subtypes of thyroid cancer and develop new effective therapeutic strategies (Zhang et al., 2019).

Vitamin D is a steroid molecule, which mainly regulates bone metabolism, calcium and phosphorus homeostasis (Makariou et al., 2011). There are two forms of vitamin D, vitamin D_3_ (cholecalciferol) and vitamin D_2_ (ergocalciferol) (Prietl et al., 2013). Both forms of vitamin D are transported to the liver where they are converted to 25-hydroxyvitamin D (25(OH) D or calcidiol) by 25-hydroxylase (CYP27A1 and CYP2R1). 25(OH) D is the major circulating and stored form of vitamin D, and its serum level is considered the best marker for assessment of vitamin D status. 25(OH) D is biologically inactive and is converted to the biologically active form 1,25-dihydroxyvitamin D (1,25(OH)2D or calcitriol) by 1α-hydroxylase (CYP27B1) in the kidneys (Kmie’c and Sworczak, 2015). 1,25(OH)2D decreases the cellular proliferation of both normal cells and cancer cells, as it regulates multiple signaling pathways involved in cellular proliferation, differentiation, apoptosis, inflammation, angiogenesis, invasion and metastasis. Recent studies indicate that vitamin D also regulates microRNA expression and may influence cancer stem cell biology (Feldman et al., 2014). in addition, it is considered as a potent immunomodulator (Holick, 2007).

Platelet-derived growth factor (PDGF) is a potent mitogen, it plays a crucial role in the formation of new blood vessels, alveoli, and intestinal villi as well as the proliferation of oligodendrocytes in the central nervous system. It has a functional role during inflammation and wound healing (Deuel and Huang, 1984; Li et al., 2007). In addition, it was found that increased expression of PDGF and its receptors associated with different disorders such as glioblastomas, breast cancer and tumors of the gastrointestinal tract (Östman and Heldin, 2007). It has been demonstrated that PDGF-AA and PDGF-α receptors are highly expressed in follicular and papillary thyroid carcinoma cell lines, as well as the presence of β-receptors in human ATC (Chen et al., 2006; Heldin et al., 1988). Those findings indicated the important effect of the aberrant expression of PDGF and its receptors in cell proliferation and carcinogenesis of TC. However, their roles in the benign diseases of the thyroid gland are still unclear (Malkomes et al., 2011). 

Insulin-like growth factor (IGF) is an important signaling pathway for regulating cell proliferation, differentiation and apoptosis. Also it has a role in promoting tumor development and progression (Swisshelm et al., 1995). Several studies have reported increased expression of IGF ligands and receptors in the tumors of breast, lung, pancreas, colon, prostate, ovary, and thyroid and it is usually associated with a poor prognosis (Bowers et al., 2015; Simpson et al., 2017).

To minimize over-treatment of indolent thyroid neoplasms, attempts to use serological and molecular tests are being developed, to help in discrimination of benign and malignant lesions. These tests can rely on alterations observed at the level of a gene or protein expression. The cooperative multidisciplinary management team and the use of additional diagnostic tools can minimize the diagnostic errors in suspicious cases. Thus the aim of the current study was to assess the serum levels of 25(OH)D, IGF-1 and PDGF in patients with benign thyroid nodules and patients with PTC in comparison to normal healthy control group. This may help us to investigate their roles in the pathogenesis and progression of thyroid cancer. In addition, it may open a new avenue for the diagnosis of thyroid disease, and could be a target for TC therapy. 

## Materials and Methods

This prospective cohort study included 190 individuals who were divided into 70 patients with histopathologically confirmed papillary thyroid cancer (PTC), 60 patients with benign thyroid nodules (BN) compared to 60 healthy controls (age and sex matched ones). Patients were presented at National Cancer Institute (NCI) and Kasr Al Ainy Faculty of Medicine, Cairo University during the period from January 2017 to December 2018. 

**Figure 1:**
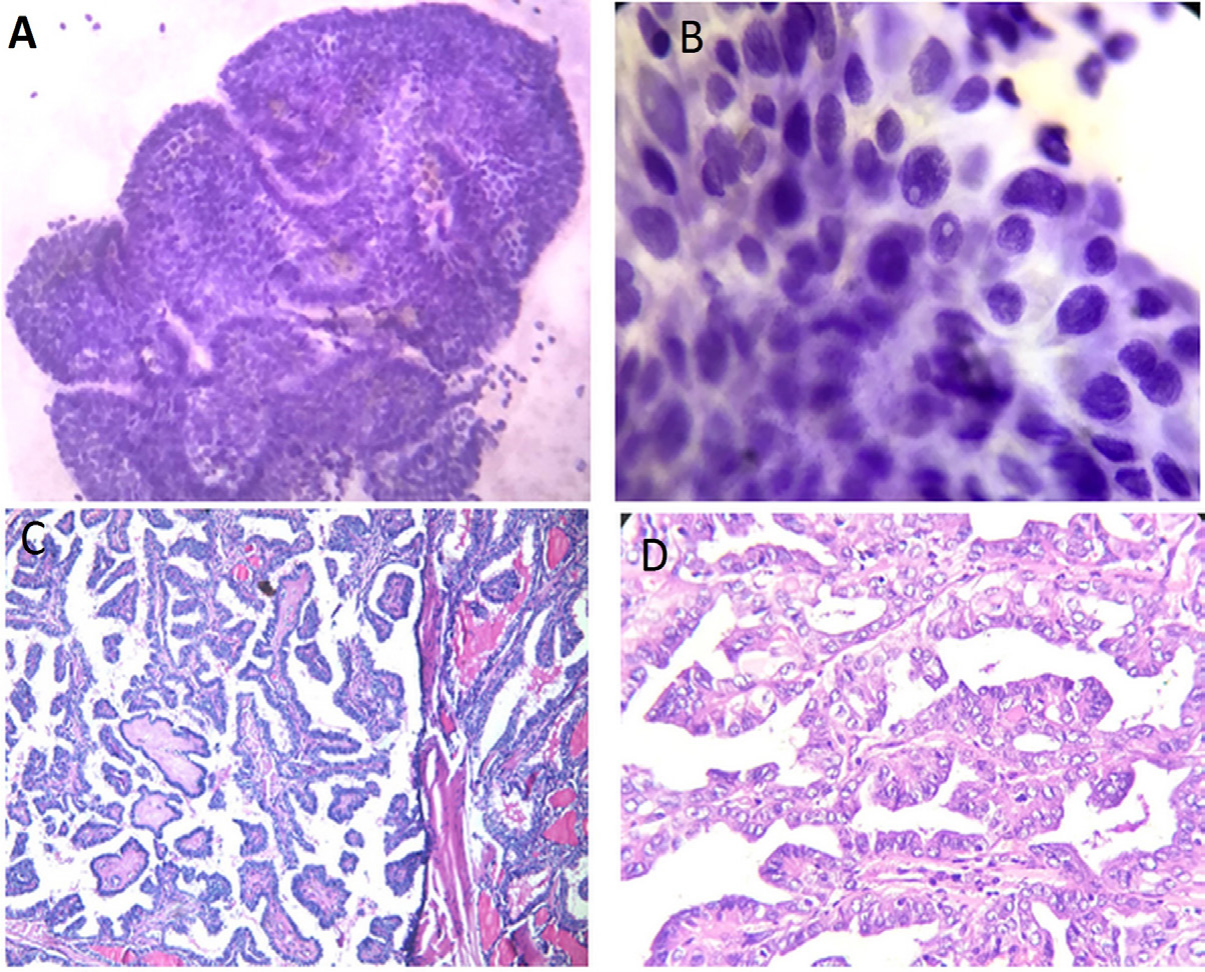
Papillary Thyroid Carcinoma Cytology. A, Papillary cellular fronds with palisading borders (Pap stain, original power 100x); B, Enlarged elongated nuclei with nuclear inclusions, focal grooving and abundant squamoid cytoplasm (Pap stain, original power 400x). Papillary thyroid carcinoma histopathology; C&D, Papillary fronds with thin fibrovascular cores. The nuclei are crowded, overlapping with clear nuclear chromatin and frequent grooving (H&E stain, original powers C- 100x & D- 200x).

All patients were subjected to full history taking, clinical and serological examination including serum free tri-iodothyronine (free T3), free thyroxin (free T4), thyroid stimulating hormone (TSH), thyroglobulin, Complete blood picture, Kidney function tests, Random blood sugar and liver enzymes assessment. 

patients with suspicious ultra-sonographic (U/S) features of TC were eligible for fine needle aspiration cytology (FNAC) according to the criteria of American thyroid association (ATA) 2015 (Haugen et al., 2016). Signs of increased risk of malignancy by U/S included hypo-echogenicity, micro- or interrupted rim calcifications, irregular Margins, absence of Halo or incomplete halo, increased intra-nodular blood flow, increase antro-posterior diameter, significant increase in size over time, invasion of anterior strap muscles, and presence of abnormal cervical lymphadenopathy. All specimens were obtained under U/S guidance using a 21- gauge needle. Samples were collected from three different sites of the targeted nodule. In case of cystic or complex nodule, the aspirated fluid of the cyst was first centrifuged in the cytopathology unit. Then it was immediately thin evenly smeared, wet fixed in 95% ethyl alcohol for minimum 15 minutes, and stained with modified Papanicolaou stain for routine cytological evaluation (Figure1). The smears for each case were examined under microscope and categorized according to Bethesda System for reporting thyroid cytology (Cibas and Ali, 2017). 

Follow-up of cytologically atypical or suspicious cases was done, thyroid surgical excision specimens were received and histopathological diagnosis was performed according to protocols for histopathology reporting of thyroid cancer as issued by U.S. College of American Pathologists (CAP) and the 8thEdition, American Joint Committee on Cancer (AJCC) Staging Manual (Tuttle et al., 2017).

Patients were excluded from the study if they had previously received radio or chemo-therapy, patients with other malignancies, any atypical/suspicious case that can’t be followed up by histopathology, or patients with Hemorrhagic inadequate FNAC samples. 

The study protocol was approved by the institutional review board of NCI, Cairo University, and ethical committee of faculty of medicine which were in accordance with 2011 declaration of Helsinki. A signed informed consent was obtained from each patient before enrollment in the study.

Sample collection: blood samples (4ml) were obtained from each patient in serum separator tubes (SST) and allowed to clot for 10 to 20 minutes at room temperature. Samples were then centrifuged at 1,000 g for 10 minutes, and the serum was aliquot and stored at -20°C until use for protein evaluations.

*Assessment of 25(OH) D, PDGF and IGF-1 in patients’ groups*

Serum levels of total vitamin D: 25(OH)D, platelet derived growth factor (PDGF), and insulin like growth factor 1 (IGF-1) were assessed in all patients using the enzyme linked immunosorbent assay (ELISA), according to manufacturer’s instructions (EIA-5396, DRG international, Inc., USA) for assessment of 25(OH)D, (cloud-clone corp, US, Cat No.: SEA436Hu) for assessment of PDGF-AB, and (cloud-clone corp, US, Cat No.: SEA050Hu) for assessment of IGF-1. All procedures were performed using a microplate reader (Tecan) at 450 nm wavelength.

*Statistical methods*

The data were analyzed using a statistical software package (SPSS Inc version 22.0; Chicago, IL, USA). Non-parametric Mann-Whitney and Kruskal-Wallis test were used to compare markers expression among groups, and its association to clinico-pathological variables. Patients and their tumor characteristics were analyzed using Chi-square test. The Area under the receiver operating curve (ROC) was calculated for each marker to investigate the best cut-off level for diagnosis of thyroid cancer. Spearman correlation was used for correlation between markers. All p-values were considered statistically significant at <0.05

**Figure 2:**
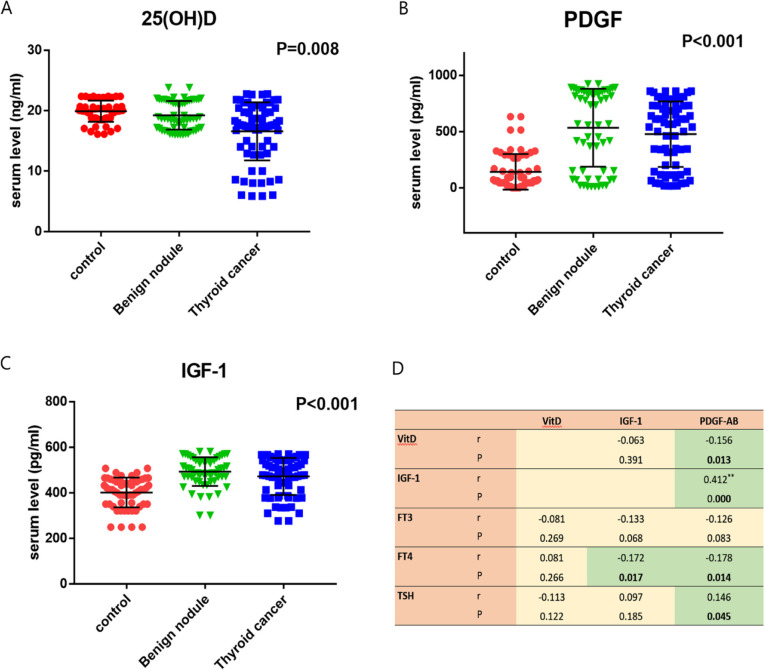
The Expression Levels of Serum. A, 25(OH)D; B, PDGF; C, IGF-1 in thyroid cancer patients, benign thyroid nodule patients and normal controls; D, correlations between the serum levels of VitD, IGF-1, PDGF in patients’ groups

**Figure 3:**
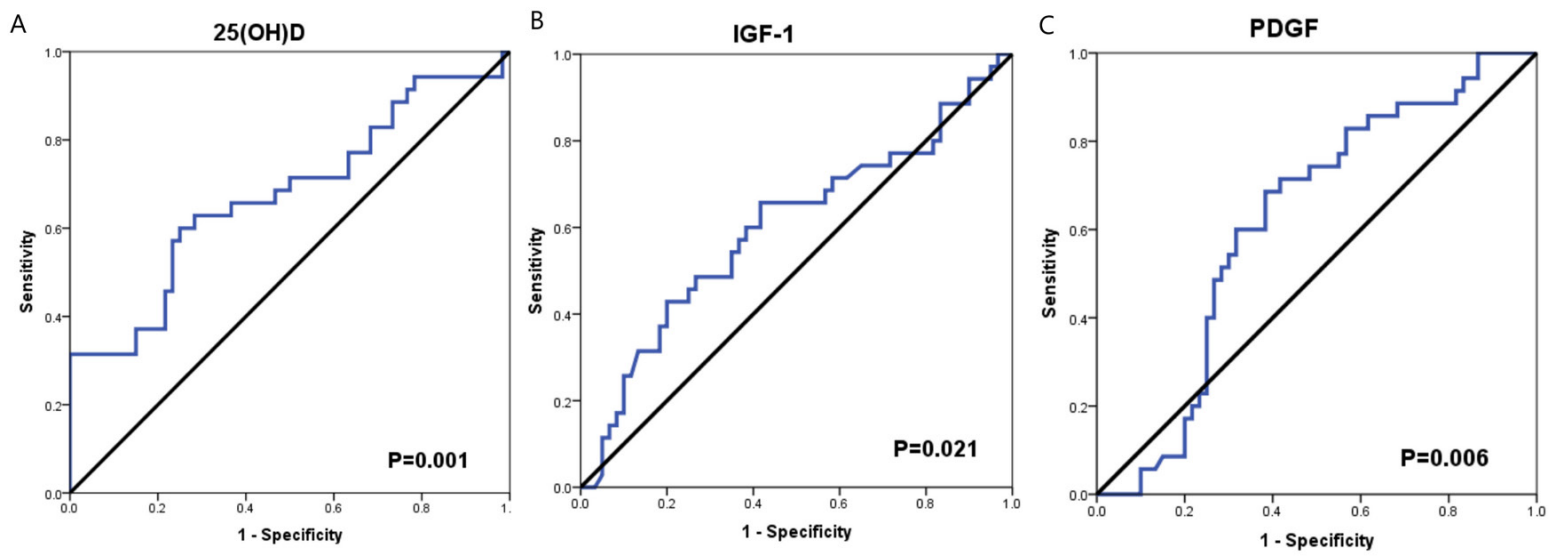
ROC Curve Analysis for 25(OH)D, IGF-1 and PDGF in Thyroid Cancer Patients

**Table 1:** Clinical Features of the Patients’ Groups

Parameter	Control group (60)	Benign nodules (60)	thyroid cancer (70)	*P*-value
Age (ys)	42 (24- 63) ^a#^	43 (27- 63) ^a^	45 (18- 65) ^a^	0.998
Gender				
Male	4 (13.3%)^a^	2 (6.7%)^a^	7 (20%)^a^	0.296
Female	26 (86.7%)	28 (93.3%)	28 (80%)	
FT3 (pg/dml)	3.1 (1.3-5.4)^a^	3 (1-5.4)^a^	2.1 (1.2- 6.1)^b^	P<0.001*
FT4 (ng/dl)	1.3 (0.5- 2.7)^a^	1.2 (0.5-10)^a^	1.1 (0.4- 20)^b^	0.008
TSH (uIU/ml)	1.5 (0.7-8.4)^a^	1.6 (0.5-8.6)^a^	28 (0.01- 279)^b^	P<0.001
25(OH)D(ng/ml)				P=0.008
Median	19.9^a^	18.7^b^	17.5^c^	
range	16.1- 22.4	16.0- 23.8	5.9-22.7	
IQR	1.88	4.63	6.47	
95% CI	19.5 – 20.4	18.6-19.8	15.4- 17.7	
PDGF (pg/ml)				P<0.001
Median	66.5^a^	652.2^b^	565.8^b^	
range	0- 632.3	10- 923.9	17.1-859.6	
IQR	222.6	704.2	588.07	
95% CI	100.7- 182.8	443.9- 622.9	408.3-547.1	
IGF-1 (pg/ml)				P<0.001
Median	414.3^a^	493.4^b^	477.8^b^	
range	249.2- 507.1	300- 580.9	276- 569.8	
IQR	98.4	73.3	130.4	
95% CI	384.4- 418.2	476.4-509.1	452.4- 491.2	

#values with similar letters are statistically insignificant. *valu

es in bold are statistically significant. 25(OH)D, total vitamin D; PDGF, platelet derived growth factor, and IGF-1: insulin like growth factor 1

**Table 2:** ROC Curve Analysis for the Diagnosis of Thyroid Carcinoma

Test Variable(s)	AUC	SE (95% CI)	cutoff	sensitivity	specificity	*P-*value
25(OH)D	0.675	0.042 (0.592- 0.758)	18.7	65.70%	63.30%	0.001*
IGF-1	0.6	0.044 (0.514- 0.687)	468.4	65.70%	58.30%	0.021
PDGF	0.619	0.041 (0.539- 0.700)	339.4	68.60%	61.70%	0.006

*values in bold are statistically significant; AUC, area under curve; SE, standard error; 25(OH)D, total vitamin D; PDGF, platelet derived growth factor; IGF-1, insulin like growth factor 1

**Figure 4:**
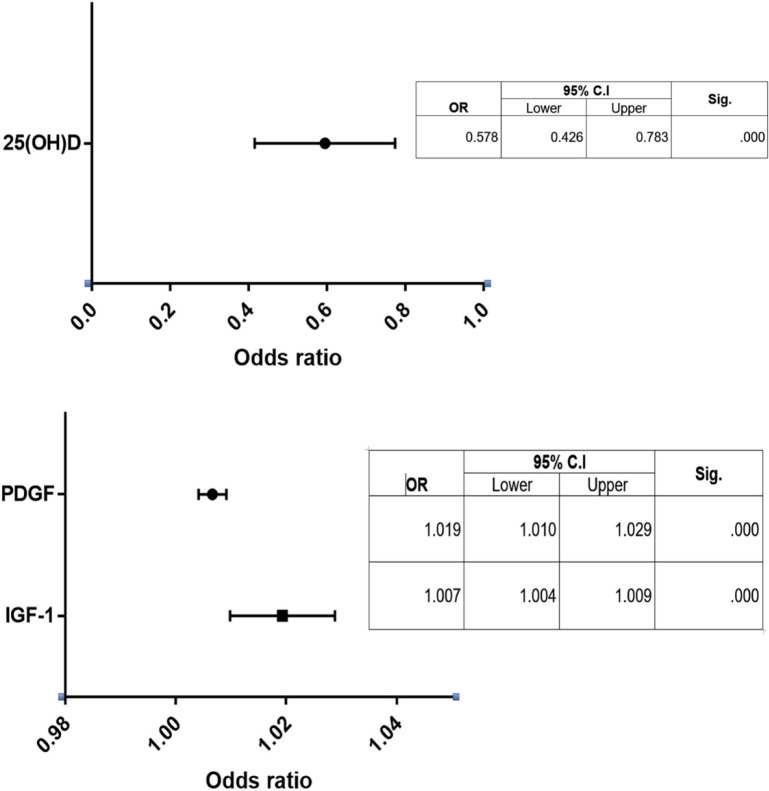
Odds Ratio with the 95% Confidence Interval for the Multivariate Regression Analysis of 25(OH)D, IGF-1 and PDGF for the Risk of Thyroid Cancer

## Results

*Patients’ characteristics*

The median age of the assessed patients’ groups was 42 (range; 24- 63) years in the control group, 43 (range; 27- 63) years in BN patients, and 45 (range; 18- 65) years in TC patients (P= 0.998). Females represented (86.7%, 93.3% and 80%) in the NC, BN and TC patients respectively (P= 0.296). Free T3 level was significantly decreased in TC patients 2.1 (1.2- 6.1), rather than in the NC and BN patients [3.1 (1.3-5.4) and 3 (1-5.4); respectively, P<0.001]. There was a significant difference in the expression levels of FT4 in the TC, NB and NC groups [1.1 (0.4- 20), 1.2 (0.5-10) and 1.3 (0.5- 2.7); respectively, P=0.008]. Also there was a significant increase in the serum level of TSH in TC patients rather than in the BN and NC patients [28 (0.01- 279), 1.6 (0.5-8.6) and 1.5 (0.7-8.4); respectively, P<0.001], Table 1.

*Assessment of serum 25(OH) D among patients’ groups*

The median and range of the serum levels of 25(OH)D is 19.9 (16.1- 22.4 ng/ml) in the control group, 18.7 (16.0- 23.8 pg/ml) in BN patients, and 17.5 (5.9-22.7 pg/ml) in TC patients (Figure 2a). There is a significant decrease in the level of 25(OH)D in TC patients compared to control group (P<0.001), and BN patients (P=0.006). Also, the level of 25(OH)D in BN patients is significantly decreased compared to control group (P=0.039, Table 1).

*Assessment of serum PDGF among patients’ groups*

The median and range of the serum levels of PDGF was 66.5 (0- 632.3 pg/ml) in the control group, 652.2 (10- 923.9 pg/ml) in BN patients, and 565.8 (17.1-859.6 pg/ml) in TC patients (Figure 2b). There was a significant increase in the serum levels of PDGF in TC patients (P<0.001), and BN patients (P<0.001) compared to the control group. However, there were no significant differences in the serum levels of PDGF between TC and BN patients (P=0.087, Table 1). 

*Assessment of serum IGF-1 among patients’ groups*

The median and range of the serum levels of IGF-1 was 414.3 pg/ml, range (249.2- 507.1 pg/ml) in the control group, 493.4 pg/ml, range (300- 580.9 pg/ml) in BN patients, and 477.8 pg/ml, range (276- 569.8 pg/ml) in TC patients (Figure 2c). There was a significant increase in the level of PDGF in TC patients (P<0.001), and BN patients (P<0.001) compared to the control group. However, there were no significant differences in the serum levels of IGF-1 between TC and BN patients (P=0.258, Table 1). 

*Correlations between the serum levels of 25(OH)D, IGF-1 and PDGF in the assessed patients*

The serum level of PDGF correlated significantly with IGF-1 (r=0.412, P<0.001), TSH (r=0.146, P=0.045), and inversely correlated with 25(OH)D (r= -0.156, P=0.013) and FT4 (r=-0.178, P=0.014). there was a significant inverse correlation between the serum levels of IGF-1 and FT4 (r=-0.172, P=0.017). on the other hand, no significant correlation was detected between the serum levels of 25(OH)D and any of the assessed markers including IGF-1, FT3, FT4 and TSH (P>0.05, Figure 2D).

*The diagnostic accuracy of serum 25(OH) D, IGF-1 and PDGF for thyroid cancer*

To evaluate the diagnostic accuracy of serum 25(OH) D, IGF-1 and PDGF for TC patients, ROC curve analysis was performed. The AUC of serum 25(OH) D was 0.675 (95% CI= 0.592- 0.758), the sensitivity and specificity were (65.7% and 63.3%; respectively, P= 0.001) at cutoff value 18.7 ng/ml. The AUC of serum IGF-1 was 0.600 (95% CI= 0.514- 0.687), the sensitivity and specificity were (65.7% and 58.3%; respectively, P=0.021) at cutoff value 468.4 pg/ml. While, the AUC of PDGF was 0.619 (95% CI= 0.539- 0.700), the sensitivity and specificity were (68.6% and 61.7%, P=0.006) at cutoff value 339.4 pg/ml (Figure 3 and Table 2). 

*Univariate and Multivariate logistic regression analysis*

Univariate logistic regression analysis showed that serum 25(OH)D, IGF-1 and PDGF were significantly associated with the risk of thyroid cancer. The Multivariate analysis also demonstrated that serum 25(OH)D (OR=0.578, 95%CI= 0.426-0.783), IGF-1 (OR=1.019, 95%CI= 1.010-1.029) and PDGF (OR=1.007, 95%CI= 1.004-1.009) were considered independent risk factors for thyroid cancer (P<0.001, for all, Figure 4). 

## Discussion

Thyroid nodules are not merely a single disease. They represent a spectrum of different thyroid diseases. The current study demonstrated that 25(OH)D is significantly decreased in TC cases compared to control group and benign nodule patients. These data are in agreement with Roskies et al., (2012), who found a higher incidence of TC in patients who had vitamin D-deficiency compared to those who had normal vitamin D levels, he suggested that vitamin D deficiency is a potentially modifiable risk factor for thyroid cancer. similarly, Sahin et al., (2013), reported that patients with PTC had significantly lower 25(OH)D levels than the control group. Moreover, Kim et al., (2014), concluded lower preoperative 25(OH)D levels associated significantly with increased tumor size >1 cm, and increased incidence of lymph node metastasis. Penna-Martinez et al., (2012), reported an association between the incidence of differentiated thyroid cancer (DTC) and low levels of 25(OH)D and 1,25(OH)2D in certain CYP24A1 haplotypes. On contrary, other studies reported no significant association between serum level of 25(OH)D and thyroid cancer risk (Jonklaas et al., 2013; Ahn et al., 2016; Danilovic et al., 2016; Kim, 2016; Choi et al., 2017). 

Another marker assessed in the current study is the serum levels of PDGF-AB in the patients’ groups, it revealed a significant increase in the level of PDGF-AB in the TC and BN patients compared to the control group. However, there were no significant differences in the serum levels of PDGF between TC and BN patients. These data are in agreement with Lopez-Campistrous et al., (2016), who demonstrated that PDGFRα promotes dedifferentiation in PTC by decreasing TTF1 expression in the nucleus, also the high level of PDGFRα could predict resistance to radioactive iodine therapy. Moreover, Ekpe-Adewuyi et al., (2016), reported that PDGFRα activation is an important mechanism that drives aggressiveness and nodal metastasis in PTC cells. He suggested that inhibition of PDGFRα could provide a potentially effective treatment for PTC patients who had PDGFRα over-expression. Another study done by Malkomes et al., (2011) demonstrated higher expression of PDGF isoforms, as well as receptors α and β concentration in thyrocytes from benign thyroid nodules compared to normal thyroid tissues. In addition, PDGF regulates the transcription of miR-146b which is associated with PTC aggressiveness and prognosis (Shao et al., 2011).

Regarding the assessment of the serum level of IGF-1 in the patients, we found that IGF-1 is significantly increased in TC and BN patients compared to the control group. However, there were no significant differences in the serum levels of IGF-1 between TC and BN patients. These data are consistent with that observed by Lawnick et al., (2020), that IGF-1 level was significantly higher in patients with PTC compared to controls. However, he found no significant increase in patients with MNG. Manzella et al., (2019), concluded that IGF axis is an important pathway for thyroid transformation, as thyroid cancer cells overexpress both IGF ligands and their receptors.

To evaluate the diagnostic accuracy of serum 25(OH) D, IGF-1 and PDGF for TC patients, ROC curve analysis was performed. We found that PDGF achieved the highest sensitivity and specificity (68.6%) and 25(OH) D achieved the highest specificity (63.3%) for the identification of thyroid cancer patients. and IGF-1, the sensitivity and specificity were (68.6% and 66.7%; respectively). For confirmation of these data, the multivariate regression analysis revealed that these markers could be an independent risk factors for thyroid cancer development.

The current study provides an evidence that serum levels of 25(OH) D, IGF-1 and PDGF are significantly different in thyroid cancer and benign nodule cases compared to normal subjects. Multivariate analysis demonstrated that serum 25(OH)D, IGF-1 and PDGF were considered independent risk factors for thyroid cancer. Hence, these markers had an important role in the pathogenesis of thyroid cancer, and could be a potential diagnostic or prognostic biomarkers for thyroid diseases. However further studies are required using larger number of patients, and assessment by different techniques to validate these results, and so it will be a target for future therapy of those patients.
